# Wide distribution of resistance to the fungicides fludioxonil and iprodione in *Penicillium* species

**DOI:** 10.1371/journal.pone.0262521

**Published:** 2022-01-31

**Authors:** Sayoko Oiki, Takashi Yaguchi, Syun-ichi Urayama, Daisuke Hagiwara

**Affiliations:** 1 Faculty of Life and Environmental Sciences, University of Tsukuba, Tsukuba, Japan; 2 Medical Mycology Research Center, Chiba University, Chiba, Japan; 3 Microbiology Research Center for Sustainability, University of Tsukuba, Tsukuba, Japan; Universita degli Studi di Pisa, ITALY

## Abstract

Fludioxonil and iprodione are effective fungicides widely used for crop protection and are essential for controlling plant pathogenic fungi. The emergence of fungicide-resistant strains of targeted pathogens is regularly monitored, and several cases have been reported. Non-targeted fungi may also be exposed to the fungicide residues in agricultural fields. However, there are no comprehensive reports on fungicide-resistant strains of non-targeted fungi. Here, we surveyed 99 strains, representing 12 *Penicillium* species, that were isolated from a variety of environments, including foods, dead bodies, and clinical samples. Among the *Penicillium* strains, including non-pathogenic *P*. *chrysogenum* and *P*. *camembertii*, as well as postharvest pathogens *P*. *expansum* and *P*. *digitatum*, 14 and 20 showed resistance to fludioxonil and iprodione, respectively, and 6 showed multi-drug resistance to the fungicides. Sequence analyses revealed that some strains of *P*. *chrysogenum* and *Penicillium oxalicum* had mutations in NikA, a group III histidine kinase of the high-osmolarity glycerol pathway, which is the mode of action for fludioxonil and iprodione. The single nucleotide polymorphisms of G693D and T1318P in *P*. *chrysogenum* and T960S in *P*. *oxalicum* were only present in the fludioxonil- or iprodione-resistant strains. These strains also exhibited resistance to pyrrolnitrin, which is the lead compound in fludioxonil and is naturally produced by some *Pseudomonas* species. This study demonstrated that non-targeted *Penicillium* strains distributed throughout the environment possess fungicide resistance.

## Introduction

Fludioxonil is a member of the phenylpyrrole class of fungicides that acts on a broad spectrum of plant pathogenic fungi [1]. It is a derivative of pyrrolnitrin, a secondary metabolite produced by certain bacteria, including *Pseudomonas* species [2] ここをクリックまたはタップしてテキストを入力してください。. In many countries, fludioxonil is widely used for crop, as well as post-harvest, protection of pom fruits from fungal pathogens. Fludioxonil’s mode of action consists of a fungal two-component system in the high-osmolarity glycerol (HOG) pathway, which is involved in major cellular responses to external stimuli, such as osmotic shock, UV irradiation, oxidative and heavy metal stresses, and high temperature [3] ここをクリックまたはタップしてテキストを入力してください。. Treatment with fludioxonil leads to an abnormal hyphal morphology, including swelling and balloon-shapes, as well as the hyperaccumulation of glycerol, and these changes have been observed in several fungal species [4, 5]. High-doses of fludioxonil produce fungicidal effects on a wide range of fungi. Thus, fludioxonil is the first choice for controlling plant pathogenic fungi in fields and for preserving harvested crops.

However, repeated applications of fungicides have resulted in the occurrence of resistant strains of pathogenic fungi [6]. Indeed, strains of *Alternaria brassicola* and *Alternaria alternata* resistant to fludioxonil have been isolated from the fields in which fludioxonil was applied [7, 8]. Molecular analyses using laboratory-derived fungicide-resistant strains have identified that mutations in a group III histidine kinase (HHK) of the HOG pathway are responsible for fludioxonil resistance [9, 10]. The resistance mechanisms of fludioxonil have been extensively studied in several fungi, including *Neurospora crassa*, *Magnaporthe oryzae*, *Botrytis cinerea*, *A*. *brassicicola*, and *Aspergillus nidulans* [11–15]. Most of the fludioxonil-resistant strains showed multi-drug resistance to iprodione, a dicarboximide fungicide, which indicates that the fungicides’ modes of action share the same target molecule.

Some mutations conferring resistance to fludioxonil and iprodione in field isolates of plant pathogens were found in group III HHKs [15–18]. Fungal HHKs are typically classified into 11 groups. The group III HHKs have a unique structure, characterized by five to seven tandem repeats of histidine kinases, adenylyl cyclases, methyl-accepting chemotaxis proteins, and phosphatases (HAMP) domains at the N-termini [19]. Owing to its essential role in many aspects of stress responses, including pathogenicity, loss-of-function mutations of the group III HHKs are thought to be maintained at a low prevalence in the field [20]. Indeed, deleting the HHK gene results in growth retardation, morphological alterations, developmental defects, and osmosensitivity [21–23], which result in higher fitness costs compared with the parental strains.

Because of the practical importance, fungicide resistance in targeted plant pathogens has been intensively investigated. However, the effects of fungicides on non-targeted fungi remain unstudied, and the ecological impact underestimated. The objective of this study was to determine whether and how non-targeted fungi acquire resistance to fludioxonil and iprodione. Thus, we searched for fludioxonil/iprodione-resistant *Penicillium* strains isolated from outside the fields and investigated whether resistant strains possessed mutations in the group III HHKs, while some targeted *Penicillium* species have been reported to show resistance to these fungicides [24, 25]. Furthermore, we examined multi-drug resistance to pyrrolnitrin among the strains and the competition among pyrrolnitrin-producing *Pseudomonas* strains.

## Materials and methods

### Strains, culture conditions, and reagents

In total, 80 *Penicillium* strains were provided through the National Bio-Resource Project, Japan (http://www.nbrp.jp/) and are preserved at the Medical Mycology Research Center, Chiba University, and 19 *Penicillium* strains were obtained from Biological Resource Center, National Institute of Technology and Evaluation (NBRC). *Penicillium* strains were cultured on potato dextrose agar (PDA) or in potato dextrose broth at 25°C for 5 days. Conidial suspension were prepared by scraping colony surfaces with a spreader and 0.05% Tween 20. The amount of conidia retrieved was counted using a hemocytometer. Pyrrolnitrin (from *Pseudomonas cepacia*) was commercially obtained (Sigma-Aldrich Co., St. Louis, MO). Fludioxonil and iprodione were obtained from the Abe laboratory at Tohoku University, Sendai, Miyagi, Japan. Oligonucleotides were synthesized by Eurofins Genomics (Tokyo, Japan).

### Antifungal susceptibility assay

Sensitivity to fungicides was determined by measuring colony growth on PDA plates in the presence of fungicides. Approximately 10,000 spores of each *Penicillium* strain were inoculated onto both PDA and PDA supplemented with 1 μg/mL fludioxonil, 10 μg/mL iprodione, or 0.05 μg/mL pyrrolnitrin and incubated at 25°C for 5 days. The diameter of each fungal colony on PDA amended with fungicides was measured and compared with that on PDA alone. Strains having a growth rate of 50% or more were defined as “fungicide-resistant”, whereas those having a growth rate of less than 50% were defined as “fungicide-sensitive”.

### Extraction of genomic DNA

Mycelia cultured in potato dextrose broth were frozen in liquid nitrogen and ground to a fine powder using a mortar and pestle. Total genomic DNA was extracted using a NucleoSpin Plant II Kit (Takara Bio, Ohtsu, Japan).

### DNA sequencing

The genes encoding NikA (500 bp-upstream of the open reading frame to 500 bp-downstream of the open reading frame) in *Penicillium chrysogenum* and *Penicillium oxalicum* were amplified by PCR using genomic DNA as the template and specific primers (S1 Table). The PCR conditions were as follows: 30 cycles of 98°C for 10 s, 53°C for 5 s, and 68°C for 1 min with KOD One PCR Master Mix (Toyobo, Osaka, Japan). The PCR product was subject to agarose gel electrophoresis and purified using a Gel/PCR Extraction Kit (NIPPON Genetics, Tokyo, Japan). The purified PCR products were subjected to DNA sequencing (Eurofins Genomics). The sequences were compared with those of the *nikA* genes from the reference genomes of *P*. *chrysogenum* P2niaD18 (GCA_000710275) and *P*. *oxalicum* 114–2 (GCA_000346795), which were retrieved from the National Center for Biotechnology Information database (https://www.ncbi.nlm.nih.gov/).

### Genome sequencing

Whole-genome sequencing using next-generation methods was performed as described previously [26]. Briefly, we prepared a fragmented DNA library from the genomic DNA of *P*. *roqueforti* using NEBNext Ultra II FS DNA Library Prep Kit for Illumina (New England BioLabs) and NEBNext Multiplex Oligos for Illumina (New England BioLabs). Paired-end sequencing was carried out by Novogene.

### Single nucleotide variant detection

To search for single nucleotide polymorphisms in *nikA* of *P*. *roqueforti*, we performed read mapping using CLC Genomics Workbench (CLC bio, Aarhus, Denmark). The reads from each isolate were trimmed and mapped to the *nikA* (PROQFM164_S03g000214) of *P*. *roqueforti* FM164 (GCA_000513255).

## Results

### Fludioxonil- and/or iprodione-resistant *Penicillium*

**Fig 1:**
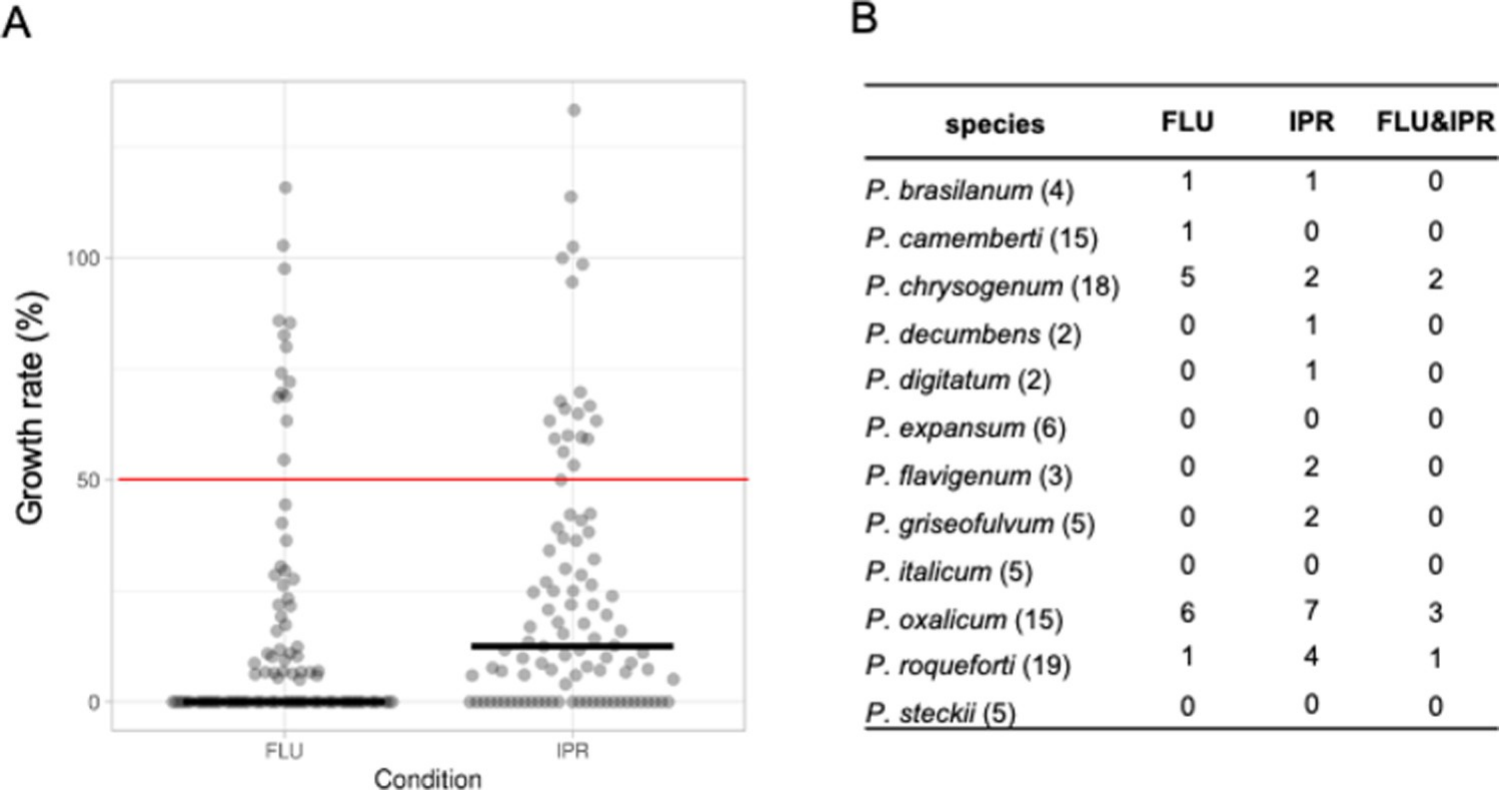
Multi-drug resistance to fludioxonil and iprodione. **(A)** Box plots showing the growth rates of 99 *Penicillium* strains in the presence of fludioxonil (FLU) and iprodione (IPR). The plots for growth rates ≥ 50% indicate resistant strains. **(B)** The number of fludioxonil and/or iprodione-resistant strains. Numbers in parentheses indicate the total number of strains.

To understand the distribution of fludioxonil and iprodione resistance in non-targeted fungal species, we prepared a set of 99 *Penicillium* strains representing 12 species. This set contained 4 *P*. *brasilanum*, 15 *P*. *camemberti*, 18 *P*. *chrysogenum*, 2 *P*. *decumbens*, 2 *P*. *digitatum*, 6 *P*. *expansum*, 3 *P*. *flavigenum*, 5 *P*. *griseofulvum*, 5 *P*. *italicum*, 15 *P*. *oxalicum*, 19 *P*. *roqueforti*, and 5 *P*. *steckii* strains (Table 1). The isolation source was registered as unknown for 17, cheese for 25, dead body for 10, patient for 13, fruit for 6, other foods for 4, soil for 6, other creatures for 3, and other environments for 15 strains (Table 1). These strains were subjected to antifungal susceptibility assays using fludioxonil and iprodione. Colony growth on PDA containing 1 μg/mL fludioxonil or 10 μg/mL iprodione was examined (S1 and S2 Figs). The concentrations of the fungicides were determined with reference to other papers describing fungicide-resistant strains. A total of 14 strains (*P*. *brasilanum*, 1/4; *P*. *camemberti*, 1/15; *P*. *chrysogenum*, 5/18; *P*. *oxalicum*, 6/15; and *P*. *roqueforti*, 1/19) and 20 strains (*P*. *brasilanum*, 1/4; *P*. *chrysogenum*, 2/18; *P*. *decumbens*, 1/2; *P*. *digitatum*, 1/2; *P*. *flavigenum*, 2/3; *P*. *griseofulvum*, 2/5; *P*. *oxalicum*, 7/15; and *P*. *roqueforti*, 4/19) showed resistance to fludioxonil and iprodione, respectively (Table 1 and Fig 1). Interestingly, six strains (two strains of *P*. *chrysogenum*, three strains of *P*. *oxalicum*, and one strain of *P*. *roqueforti*) exhibited multi-drug resistance to fludioxonil and iprodione. No strains resistant to either of the fungicides were present in *P*. *expansum*, *P*. *italicum*, or *P*. *steckii*.

**Table 1:** The *Penicillium* strains used in this study.

No	Species	Strain ID	FLU^a^	IPR^b^	PRN^c^	Source
1	*P*. *brasilanum*	IFM 42067 (= IFO 6234)	S	S	R	soil
2	*P*. *brasilanum*	IFM 42077	S	R	R	unknown
3	*P*. *brasilanum*	IFM 60071	S	S	R	environmental isolate
4	*P*. *brasilanum*	IFM 60072	R	S	R	environmental isolate
5	*P*. *camemberti*	IFM 49450 (= CBS 299.48)	S	S	R	French Camembert cheese
6	*P*. *camemberti*	IFM 54179	S	S	R	environmental isolate
7	*P*. *camemberti*	IFM 61933	S	S	R	tongue (dead body)
8	*P*. *camemberti*	NBRC 5855	S	S	R	unknown
9	*P*. *camemberti*	NBRC 32215	S	S	S	Commercial cheese
10	*P*. *camemberti*	NBRC 105299	S	S	R	Camembert cheese imported from France
11	*P*. *camemberti*	NBRC 105301	R	S	R	Camembert cheese imported from France, Japan
12	*P*. *camemberti*	NBRC 105305	S	S	S	Lys bleu cheese imported from France
13	*P*. *camemberti*	NBRC 105306	S	S	S	Bonifaz cheese imported from Germany, Japan
14	*P*. *camemberti*	NBRC 105307	S	S	S	Bonifaz cheese imported from Germany
15	*P*. *camemberti*	NBRC 105308	S	S	S	Cambozola cheese imported from Germany
16	*P*. *camemberti*	NBRC 105309	S	S	S	Cambozola cheese imported from Germany, Japan
17	*P*. *camemberti*	NBRC 105310	S	S	S	Bavariablu cheese imported from Germany
18	*P*. *camemberti*	NBRC 105314	S	S	S	Natural cheese made in Hokkaido, Japan
19	*P*. *camemberti*	NBRC 105315	S	S	S	Camembert cheese made in Japan
20	*P*. *chrysogenum*	IFM 40614	S	S	S	unknown
21	*P*. *chrysogenum*	IFM 47464 (= CBS 349.48)	R	S	R	unknown, UK
22	*P*. *chrysogenum*	IFM 47768	S	S	S	unknown, Japan
23	*P*. *chrysogenum*	IFM 52203	S	S	S	bathroom, Brazil
24	*P*. *chrysogenum*	IFM 52204	S	S	S	kitchen, Brazil
25	*P*. *chrysogenum*	IFM 56829	S	S	S	50 man, China
26	*P*. *chrysogenum*	IFM 57112	S	S	S	bioresource
27	*P*. *chrysogenum*	IFM 57243 (= CBS 282.97)	R	R	R	dust from school, Denmark
28	*P*. *chrysogenum*	IFM 57244 (= CBS 798.97)	R	R	R	Apeldoorn / indoor environment, Netherland
29	*P*. *chrysogenum*	IFM 57245 (= CBS 478.84)	R	S	R	air, fruit store, Denmark
30	*P*. *chrysogenum*	IFM 59766	S	S	S	buttock (dead body)
31	*P*. *chrysogenum*	IFM 60605	R	S	R	skin of jaw (dead body)
32	*P*. *chrysogenum*	IFM 60953	S	S	S	right finger (dead body)
33	*P*. *chrysogenum*	IFM 61615	S	S	S	swab from patient’s house
34	*P*. *chrysogenum*	IFM 61632	S	S	S	face (dead body)
35	*P*. *chrysogenum*	IFM 62336	S	S	S	trachea (dead body)
36	*P*. *chrysogenum*	IFM 63007	S	S	R	right leg (dead body)
37	*P*. *chrysogenum*	IFM 64696	S	S	S	breast bone (dead body)
38	*P*. *decumbens*	IFM 46582	S	R	R	contaminant of Sporotrichosis patient
39	*P*. *decumbens*	IFM 63512	S	S	R	bedroom
40	*P*. *digitatum*	IFM 60598	S	R	S	lemon
41	*P*. *digitatum*	IFM 63755	S	S	R	62 F, sputum, ABPM
42	*P*. *expansum*	IFM 40618	S	S	S	unknown
43	*P*. *expansum*	IFM 47463 (= CBS 325.48)	S	S	S	fruit of Malus syvestris
44	*P*. *expansum*	IFM 47478 (= IFO 8800)	S	S	S	unknown, Patulin production
45	*P*. *expansum*	IFM 52210	S	S	S	nursing room, Brazil
46	*P*. *expansum*	IFM 58916	S	S	S	unknown, cyclopiazone acid production
47	*P*. *expansum*	IFM 62049	S	S	S	refrigerator
48	*P*. *flavigenum*	IFM 54184 (= CBS 110406)	S	S	S	soil under *Chrysothamnus nauseosus*
49	*P*. *flavigenum*	IFM 54185 (= CBS 110407)	S	R	R	white beans
50	*P*. *flavigenum*	IFM 54186 (= CBS 419.89)	S	R	S	flour
51	*P*. *griseofulvum*	IFM 42069 (= IAM 7212)	S	S	S	unknown
52	*P*. *griseofulvum*	IFM 47730 (= IFO 7640)	S	S	S	unknown, Belgium
53	*P*. *griseofulvum*	IFM 47791 (= CBS 124.14)	S	R	R	soil, UK
54	*P*. *griseofulvum*	IFM 54187	S	S	R	unknown
55	*P*. *griseofulvum*	IFM 54314	S	R	S	soil
56	*P*. *italicum*	IFM 49452 (= CBS 719.73)	S	S	S	fruit of Citrus sp., Israel
57	*P*. *italicum*	IFM 49453 (= CBS 339.48)	S	S	S	fruit of Citrus sp., USA
58	*P*. *italicum*	IFM 52160	S	S	S	orpharyngeal swab, Brazil
59	*P*. *italicum*	IFM 53256 (= NBRC 9419)	S	S	S	fruit of Satsuma orange
60	*P*. *italicum*	IFM 59474	S	S	S	orange of NZ, isolated in Japan
61	*P*. *oxalicum*	IFM 49446 (= CBS 219.30)	S	S	S	soil, USA
62	*P*. *oxalicum*	IFM 54751	R	S	R	enironmental isolate
63	*P*. *oxalicum*	IFM 55886	S	S	R	soil, China
64	*P*. *oxalicum*	IFM 57073	S	R	S	garbage
65	*P*. *oxalicum*	IFM 59246	R	R	R	skin (Trichechus manatus)
66	*P*. *oxalicum*	IFM 60000	S	S	S	skull (dead body)
67	*P*. *oxalicum*	IFM 61428	S	S	S	BALF, drowning
68	*P*. *oxalicum*	IFM 62137	S	R	R	bean sprouts
69	*P*. *oxalicum*	IFM 62827	S	R	R	92 F, cornea
70	*P*. *oxalicum*	IFM 62922	R	S	R	49 M, tracheal mucus plug
71	*P*. *oxalicum*	IFM 62931	S	S	S	62 M, washing solution (lung)
72	*P*. *oxalicum*	IFM 62937	R	R	R	72 M, left lung apex cavity, simple pulmonary aspergilloma
73	*P*. *oxalicum*	IFM 63612	R	R	R	68 F, BALF
74	*P*. *oxalicum*	IFM 63698	R	S	R	12 F, sputum
75	*P*. *oxalicum*	IFM 65074	S	R	S	54 F, sputum
76	*P*. *roqueforti*	IFM 47733 (= IFO 4622)	S	S	S	requefort cheese
77	*P*. *roqueforti*	IFM 48062	S	S	S	Blue-veained cheese (Gorgonzola)
78	*P*. *roqueforti*	IFM 48063	S	S	S	Blue-veained cheese (Cambozola)
79	*P*. *roqueforti*	IFM 48064	S	S	S	Blue-veained cheese (Dana blue)
80	*P*. *roqueforti*	IFM 48065	S	S	S	Blue-veained cheese (Blue-S)
81	*P*. *roqueforti*	IFM 48066	S	S	S	Blue-veained cheese (Stilton)
82	*P*. *roqueforti*	IFM 48067	S	S	S	Blue-veained cheese (Stilton)
83	*P*. *roqueforti*	IFM 48068	S	R	S	Blue-veained cheese (Roquefort)
84	*P*. *roqueforti*	IFM 48069	S	R	S	Blue-veained cheese (Roquefort)
85	*P*. *roqueforti*	IFM 48070	S	R	S	Blue-veained cheese (Blue-S)
86	*P*. *roqueforti*	IFM 48071	S	S	S	Blue-veained cheese (Blue-H)
87	*P*. *roqueforti*	IFM 58915	S	S	S	unknown, cyclopiazone acid production
88	*P*. *roqueforti*	NBRC 4622	S	S	S	Roquefort cheese
89	*P*. *roqueforti*	NBRC 5459	S	S	S	French roquefort cheese, USA
90	*P*. *roqueforti*	NBRC 5754	S	S	S	unknown
91	*P*. *roqueforti*	NBRC 5956	R	R	S	unknown
92	*P*. *roqueforti*	NBRC 6400	S	S	S	unknown
93	*P*. *roqueforti*	NBRC 7693	S	S	S	unknown
94	*P*. *roqueforti*	NBRC 8799	S	S	S	unknown
95	*P*. *steckii*	IFM 62327	S	S	S	leg (dead body)
96	*P*. *steckii*	IFM 63697	S	S	S	82 F, eye
97	*P*. *steckii*	IFM 64403	S	S	S	noodle soup
98	*P*. *steckii*	IFM 64663	S	S	S	cockroach
99	*P*. *steckii*	IFM 64664	S	S	R	cockroach

^a^ FLU indicates fludioxonil: S and R indicate sensitive and resistant, respectively.

^b^ IPR indicates iprodione.

^c^ PRN indicates pyrrolnitrin.

### Mutations in the group III HHK, NikA

**Fig 2:**
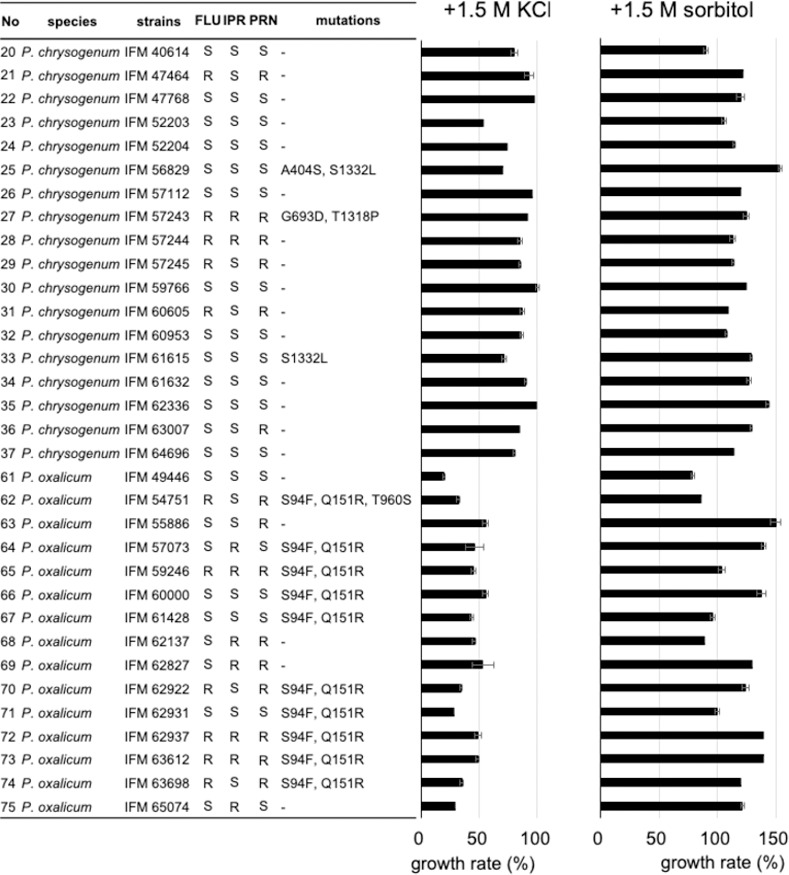
Mutations in the NikA proteins and resistance to high osmotic pressure in *P*. *chrysogenum* and *P*. *oxalicum*. The list of mutations in the NikA proteins (left) and colony growth rates in the presence of a high concentration of KCl (middle) and sorbitol (right). The data represent the averages of triplicate individual experiments (means ± standard deviations).

**Fig 3:**
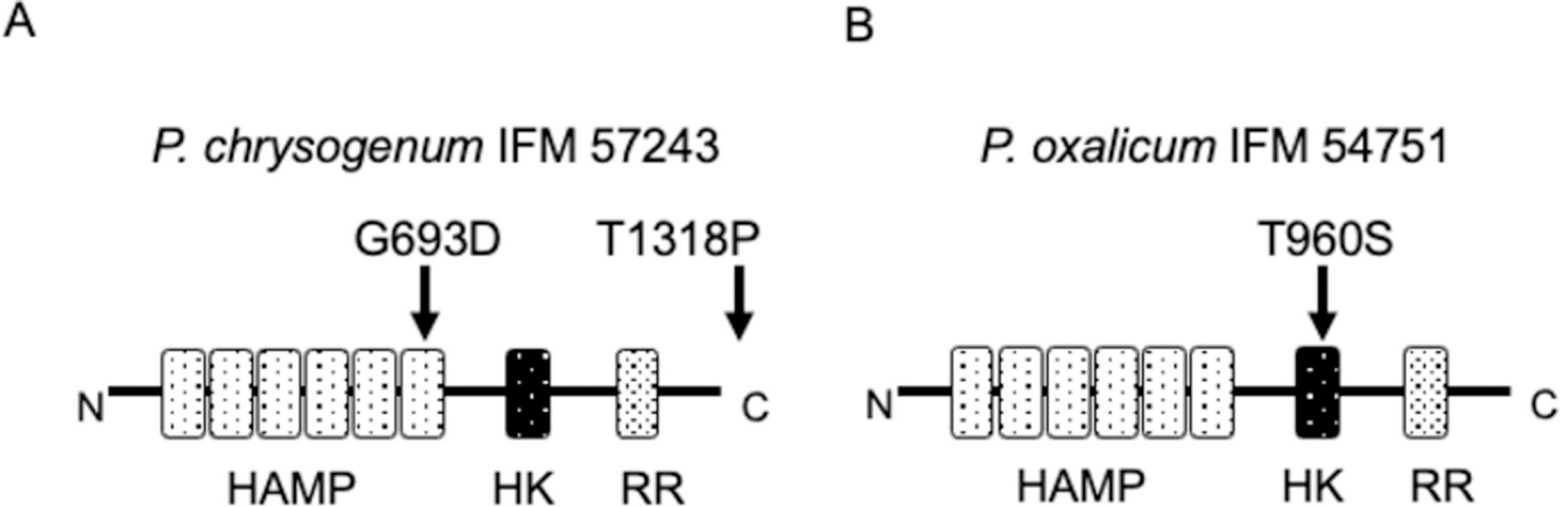
Domain structures of fungal NikA proteins. The group III HHK NikA is composed of six repeated HAMP, histidine kinase (HK), and response regulator (RR) domains. **(A)** The mutation G693D in *P*. *chrysogenum* IFM 57243 is located in the HAMP domain, whereas the mutation T1318P is located in a disordered region. **(B)** The mutation T960S in *P*. *oxalicum* IFM 54751 is located in the HK domain.

As demonstrated in several fungi, including plant pathogens, fludioxonil/iprodione-resistance might be attributed to mutations in the group III HHK, NikA, in *Penicillium* strains. In further analyses, we focused on *P*. *chrysogenum*, *P*. *oxalicum*, and *P*. *roqueforti* because they contained multiple multi-drug resistant strains. The *nikA* genes in a set of *P*. *chrysogenum*, *P*. *oxalicum* strains were sequenced and compared with those of *P*. *chrysogenum* strain P2niaD18 (GCA_000710275), *P*. *oxalicum* 114–2 (GCA_000346795), respectively. The *nikA* genes in a set of *P*. *roqueforti* strains were obtained by genome sequencing. There were several amino acid alterations in NikA (Fig 2), such as the combination of A404S and S1332L in *P*. *chrysogenum* IFM 56829. The S1332L mutation was also present in *P*. *chrysogenum* IFM 61615. Both strains were sensitive to fludioxonil and iprodione, suggesting that the mutations are not involved in the resistance. Conversely, in *P*. *chrysogenum* IFM 57243, which showed resistance to fludioxonil and iprodione, glycine was changed to aspartic acid at position 693 (G693D) and threonine was changed to proline at position 1318 (T1318P). The mutation at position 693 is located in the HAMP domain region, suggesting that this mutation affects the sensing of, and interactions with, the fungicides (Fig 3). For *P*. *oxalicum*, 10 of 15 strains harbored both S94F and Q151R mutations regardless of their fungicide-resistance level, indicating that these mutations are not associated with fungicide resistance. In *P*. *oxalicum* IFM 54751, which is resistant to fludioxonil but not to iprodione, threonine was changed to serine at position 960 (T960S) in addition to the abovementioned two mutations. This mutation is located in the kinase domain and potentially affects the histidine kinase function and fludioxonil resistance. In *P*. *roqueforti*, no amino acid alterations were found in *nikA* compared with the reference sequence.

### Resistance of *Penicillium* strains to high osmotic stress

As demonstrated in other species, the HOG pathway is involved in responses to fungicides and osmotic conditions. To test the link between resistance to fungicides and osmotic stress, we investigated the colony growth of *P*. *chrysogenum* and *P*. *oxalicum* strains on PDA containing high concentrations of KCl or sorbitol (1.5 M) (Fig 2). Among the 18 tested strains of *P*. *chrysogenum*, IFM 52203 showed a sensitivity to KCl (growth was less than 60% compared with under stress-free conditions). The growth rates of *P*. *chrysogenum* IFM 57243, which had G693D and T1318P mutations in NikA, in the presence of high concentrations of KCl and sorbitol were 92% and 124%, respectively. These values were comparable to those of other strains, indicating that the G693D and T1318P mutations had no effect on the strains’ sensitivity to osmotic stress. Compared with *P*. *chrysogenum*, *P*. *oxalicum* strains were relatively sensitive to KCl. Growth was less than 30% in the presence of KCl, compared with no KCl stress, in 3 of 15 strains, whereas only 1 strain showed >20% reduction in the colony growth in the presence of sorbitol. The growth rates of *P*. *oxalicum* IFM 54751, with of the T960S mutation in NikA, in high concentrations of KCl and sorbitol were 32% and 86%, respectively. The moderate sensitivity to high osmotic stresses suggests the involvement of the T960S mutation in osmotic stress adaptation.

### Pyrrolnitrin-resistant *Penicillium*

**Fig 4:**
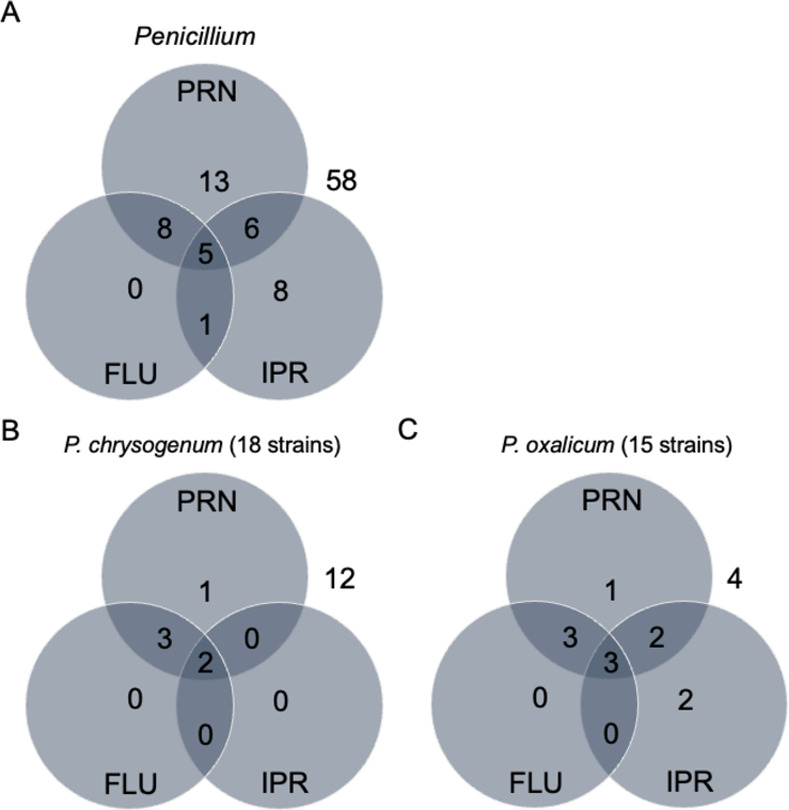
Multi-drug resistance to pyrrolnitrin, fludioxonil, and iprodione. Venn diagrams of the numbers of pyrrolnitrin-, fludioxonil-, and iprodione-resistant strains in *Penicillium* species **(A)**, *P*. *chrysogenum* **(B)**, and *P*. *oxalicum* **(C)**. PRN, pyrrolnitrin; FLU, fludioxonil; and IPR, iprodione.

Fludioxonil is an analog of the natural antifungal compound pyrrolnitrin, which is produced by some *Pseudomonas* species [27]. The mode of action for pyrrolnitrin is believed to be related to the fungal group III HKKs of the HOG pathway [28]. Therefore, we performed antifungal assays using *Penicillium* strains in the presence of pyrrolnitrin. Among the 99 tested strains of *Penicillium*, 32 (*P*. *brasilianum*, 4/4; *P*. *camemberti*, 6/15; *P*. *chrysogenum*, 6/18; *P*. *decumbens*, 2/2; *P*. *digitatum*, 1/2; *P*. *flavigenum*, 1/3; *P*. *griseofulvum*, 2/5; *P*. *oxalicum*, 9/15; and *P*. *steckii*, 1/5) showed resistance to 0.05 μg/mL pyrrolnitrin (Table 1). No strains of *P*. *expansum*, *P*. *italicum*, and *P*. *roqueforti* showed resistance to pyrrolnitrin. Notably, 13 (*P*. *brasilanum*, 1/4; *P*. *camemberti*, 1/15; *P*. *chrysogenum*, 5/18; and *P*. *oxalicum*, 6/15) and 11 (*P*. *brasilanum*, 1/4; *P*. *chrysogenum*, 2/18; *P*. *decumbens*, 1/2; *P*. *flavigenum*, 1/3; *P*. *griseofulvum*, 1/5; and *P*. *oxalicum*, 5/15) of the 32 pyrrolnitrin-resistant strains showed multi-drug resistance to fludioxonil and/or iprodione (Fig 4A). For *P*. *chrysogenum*, five and two of seven pyrrolnitrin-resistant strains showed multi-drug resistance to fludioxonil and iprodione, respectively (Fig 4B). For *P*. *oxalicum*, six and five of nine pyrrolnitrin-resistant strains showed multi-drug resistance to fludioxonil and iprodione, respectively (Fig 4C).

*Penicillium chrysogenum* IFM 57243 with G693D and T1318P mutations in NikA showed resistance to pyrrolnitrin, whereas *P*. *chrysogenum* IFM 56829 and IFM 61615 were sensitive to pyrrolnitrin. Thus, the G693D and T1318P mutations may contribute to resistance against the three fungicides. *Penicillium oxalicum* IFM 54751 with the T960S mutation exhibited resistance to fludioxonil and pyrrolnitrin. The results of the antifungal susceptibility testing revealed that within a set of *Penicillium* strains isolated from various environments that are non-targeted fungi some members show resistance to an agricultural fungicide, as well as its lead compound, which is naturally produced by bacteria in the environment.

## Discussion

In this study, we explored *Penicillium* strains resistant to the widely used fungicides fludioxonil and iprodione, as well as pyrrolnitrin. Strains of *Penicillium* species that cause postharvest decay of citrus and fruits, and exhibiting resistance to these fungicides, have been investigated [24, 25]. Interestingly, in the present study there were no resistant strains found in *P*. *expansum* and *P*. *italicum*, where one *P*. *digitatum* strain isolated from lemon showed resistance to iprodione. To the best of our knowledge, except for these pathogens, we are the first to demonstrate that several strains of non-targeted fungi, such as penicillin-producing *P*. *chrysogenum*, environmentally ubiquitous *P*. *oxalicum*, and cheese-producing *P*. *roqueforti*, show resistance to fludioxonil, iprodione, and pyrrolnitrin. A multi-drug resistance to these fungicides was detected in some strains. These results raised questions regarding the mechanisms and occurrence of resistance in these species.

**Fig 5:**
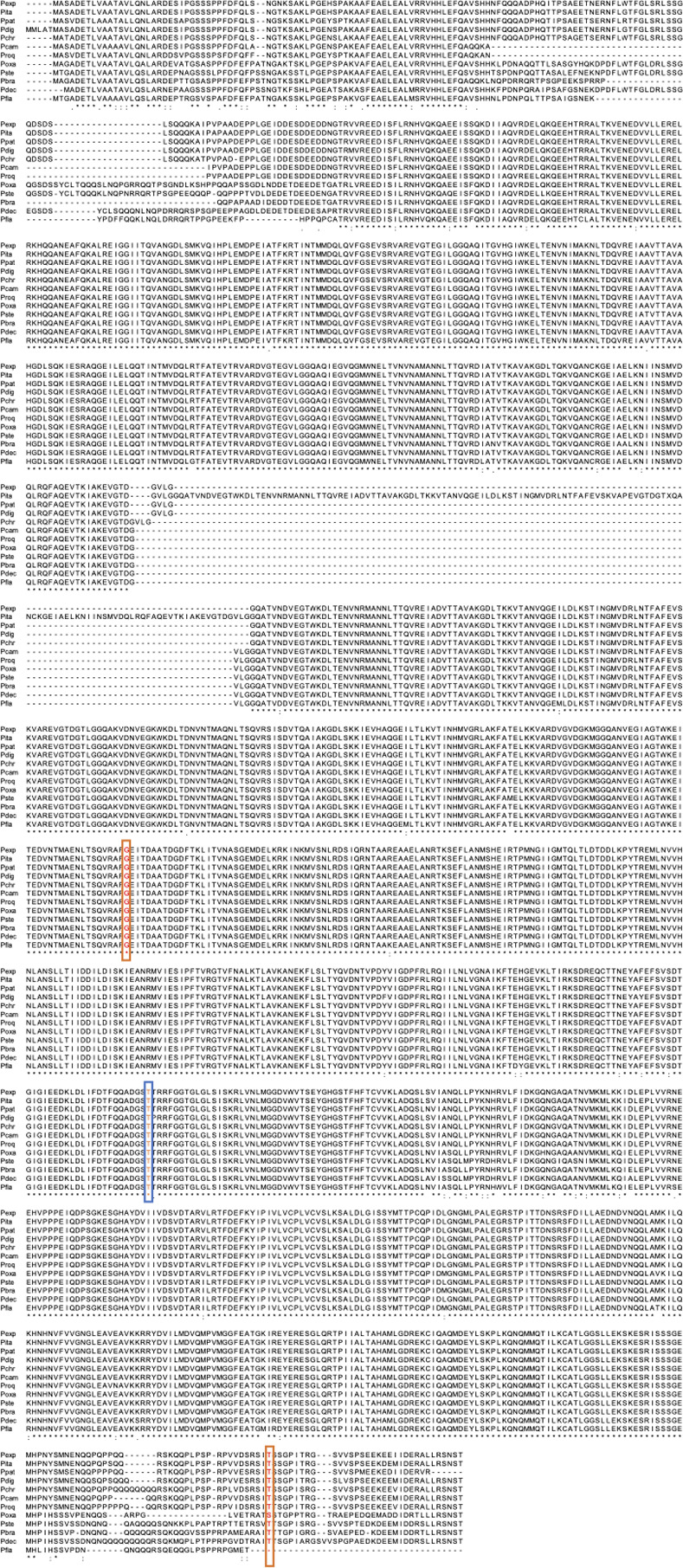
Sequence alignment of NikA in 12 *Penicillium* species. The gene IDs are as follows: *P*. *brasilanum* PMG11_02111, *P*. *camemberti* PCAMFM013_S001g000092, *P*. *chrysogenum* EN45_023640, *P*. *decumbens* PENDEC_c002G07053, *P*. *digitatum* Pdw03_4331, *P*. *expansum* PEX2_037120, *P*. *flavigenum* PENFLA_c003G00500, *P*. *griseofulvum* PGRI_040000, *P*. *italicum* PITC_092520, *P*. *oxalicum* PDE_05313, *P*. *roqueforti* PROQFM164_S03g000214, and *P*. *steckii* PENSTE_c014G10375. The amino acid residues framed by orange and blue show the mutations detected in *P*. *chrysogenum* IFM 57243 and *P*. *oxalicum* IFM 54751, respectively.

The mechanisms underlying resistance to fludioxonil have been studied in several fungi, including plant pathogens such as *B*. *cinerea*, *Cochliobolus heterostrophus*, and *M*. *oryzae* [29–31]. The fungal HOG pathway involved in osmotic stress adaptation is a target of these fungicides [10, 23, 32]. Laboratory-generated mutants resistant to these fungicides provide clear perspectives on the mechanisms. Fillinger et al. showed that exposure to pyrrolnitrin or iprodione results in resistant *B*. *cinerea* mutants, most of which harbor de novo mutations in the Bos1 protein, a group III HHK of the fungus [33]. The mutations occurred in the protein’s six repeated HAMP domains. The regeneration of site-directed clones clarified that the amino acid alterations in the HAMP domains are responsible for the fungicide resistance. Some mutations resulted in fungicide resistance and hypersensitivity to osmotic stress, whereas other mutations resulted in resistance to iprodione, but not to phenylpyrroles, and sensitivity to hyperosmolarity. Here, some *Penicillium* strains possessed mutations in NikA that were associated with fungicide resistance. *Penicillium chrysogenum* IFM 57243 showed multi-drug resistance to fludioxonil and iprodione, as well as pyrrolnitrin, and had G693D and T1318P mutations in the NikA protein. *Penicillium oxalicum* IFM 54751 showed resistance to fludioxonil and pyrrolnitrin, but not to iprodione, and had a T960S mutation in the NikA protein. These mutations are located in the amino acid residues highly conserved among the 12 species (Fig 5). These mutations may contribute to antifungal compound resistance, while site-directed clones of the mutations need to be created in the future.

A fitness penalty has been reported in fludioxonil-resistant isolates of plant pathogens, as indicated by their relatively slower mycelial growth rates or decreased pathogenicity levels [15, 34, 35] might explain the decreased fitness levels of fludioxonil-resistant strains in the field. Interestingly, the fungicide-resistant *Penicillium* strains found in the present work showed no apparent growth defects on PDA compared with sensitive isolates of the same species (S1–S3 Figs). This suggested that *Penicillium* species pay almost no fitness costs for phenylpyrrole and dicarboximide resistance, which should be investigated further.

Here, several strains of *P*. *chrysogenum* and *P*. *oxalicum* without mutations in the NikA showed resistance to one of the antifungal compounds. In *N*. *crassa*, the components of the HOG pathway, which function downstream of the group III HKK Os1, are responsible for fungicide resistance [21, 36]. Indeed, a strain with a mutation in the *os2* gene, which encodes a mitogen-activated protein (MAP) kinase in the HOG pathway, shows resistance to the fungicides. However, a mutant of the SakA MAP kinase, which is an ortholog of Os2, in *A*. *nidulans* shows only slight resistance to fludioxonil and iprodione [23]. The HOG pathway contributes to fungicide responses in different ways among fungal species. To date, only one study has investigated the HOG pathway’s role in the fungicide responses of *Penicillium*. Wang et al. demonstrated that the gene deletion mutant of Pdos2, which encodes an Os2 MAP kinase, constructed in *P*. *digitatum* shows only slight resistance to fludioxonil and iprodione, suggesting that its HOG pathway has a limited impact on fungicide sensitivity [37]. Thus, it is unlikely that fungicide sensitivity can be attributed to mutations in HOG pathway components, because the fungicide resistance levels identified here were relatively high. The resistance mechanisms of fludioxonil and iprodione are poorly understood, and thus uncharacterized mutations may be present in the resistant strains. More comprehensive investigations are needed to fully understand how the non-targeted fungi possess resistance to synthetic fungicides.

The *Penicillium* strains used in this study were collected from diverse environmental and clinical sources. According to their records, they have no history of phenylpyrrole or dicarboximide fungicide exposure. The growth test indicated that each species was naturally susceptible, and some strains acquired resistance, to the fungicides. However, it is not known where and how these *Penicillium* strains have become resistant. One plausible cause for resistance is exposure to fungicide residues in environmental organic matters, such as plant litter or compost. Fludioxonil and iprodione are registered as fungicides for use on a wide variety of crops, and thus, huge amounts of plant debris contaminated with residual fungicides are generated in agricultural settings. Ubiquitously present non-targeted *Penicillium* strains may encounter such environments, resulting in their being placed under fungicide pressure. This might lead to the natural occurrence of resistance. Another possibility is exposure to environmental pyrrolnitrin produced by certain bacteria in the environment. Pyrrolnitrin is a lead compound of phenylpyrroles, and multi-drug resistance between pyrrolnitrin and phenylpyrroles has been reported [38]. Many bacterial species that belong to the genera *Burkholderia* and *Pseudomonas* produce pyrrolnitrin [39]. Some strains have been isolated from the rhizosphere and used as biological control agents against plant pathogenic fungi in agriculture. Therefore, there might be several environmental niches having high concentrations of microbial pyrrolnitrin. Thus, non-targeted *Penicillium* strains may have acquired resistance to pyrrolnitrin and multi-drug resistance to fludioxonil/iprodione owing to exposure to pyrrolnitrin produced by indigenous species. This study warns of the potential risk of non-targeted fungi around the world acquiring resistance to the fungicides. This issue requires further clarification.

## Supporting information

S1 Fig Resistance to fludioxonil. 99 *Penicillium* strains grown in PDA (left, CTRL) and PDA containing 1 μg/mL fludioxonil (right, FLU). Colony growth rate in the presence of fludioxonil ≥ 50%; “fungicide-resistant”, < 50%; “fungicide-sensitive”.
(TIFF)

S2 Fig Resistance to iprodione. 99 *Penicillium* strains grown in PDA (left, CTRL) and PDA containing 10 μg/mL iprodione (right, IPR). Colony growth rate in the presence of iprodione ≥ 50%; “fungicide-resistant”, < 50%; “fungicide-sensitive”.
(TIFF)

S3 Fig Resistance to pyrrolnitrin. 99 *Penicillium* strains grown in PDA (left, CTRL) and PDA containing 0.05 μg/mL pyrrolnitrin (right, PRN). Colony growth rate in the presence of pyrrolnitrin ≥ 50%; “fungicide-resistant”, < 50%; “fungicide-sensitive”.
(TIFF)

S1 Table PCR primers used in this study. (DOCX)
