# Supplementary figures and images for: Wide distribution of resistance to the fungicides fludioxonil and iprodione in Penicillium species
Source: PLoS One. 2022 Jan 31;17(1):e0262521. doi: 10.1371/journal.pone.0262521 (PMC8803201; doi:10.1371/journal.pone.0262521)

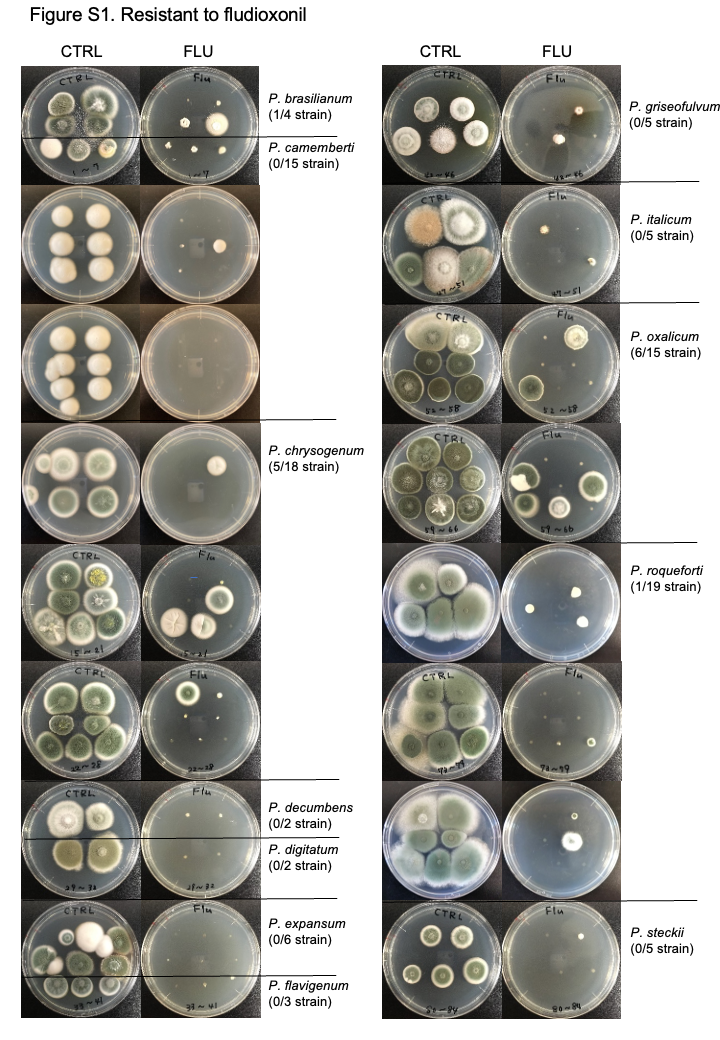

Supplement: S1 Fig — 99 Penicillium strains grown in PDA (left, CTRL) and PDA containing 1 μg/mL fludioxonil (right, FLU). Colony growth rate in the presence of fludioxonil ≥ 50%; “fungicide-resistant”, < 50%; “fungicide-sensitive”. (TIFF) [file pone.0262521.s001.tiff]

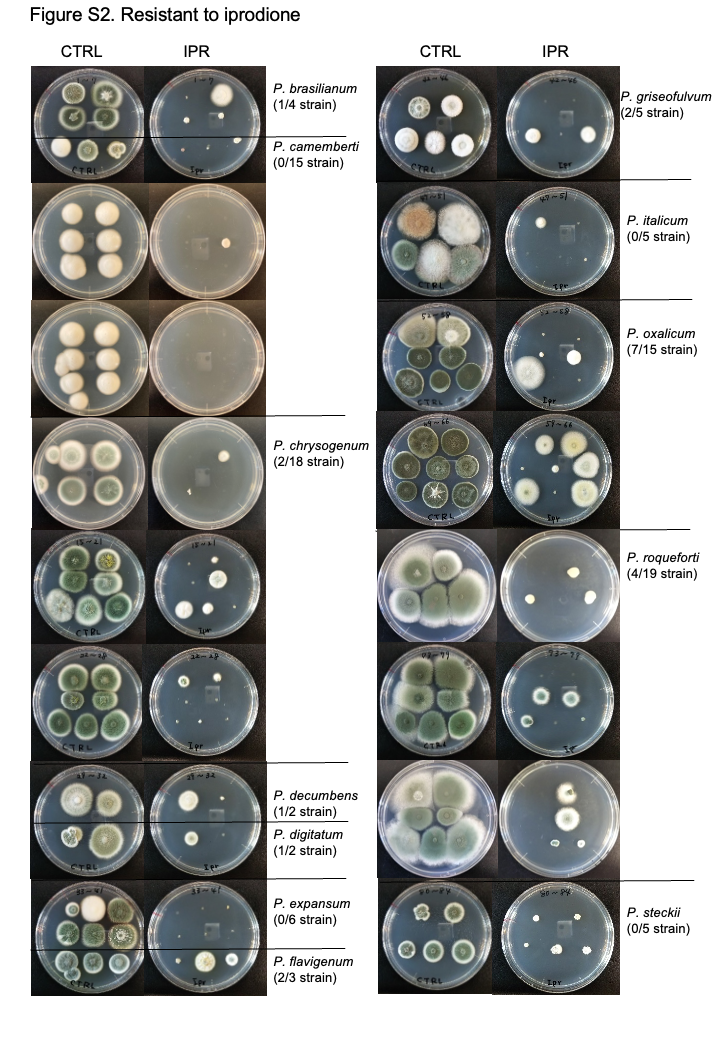

Supplement: S2 Fig — 99 Penicillium strains grown in PDA (left, CTRL) and PDA containing 10 μg/mL iprodione (right, IPR). Colony growth rate in the presence of iprodione ≥ 50%; “fungicide-resistant”, < 50%; “fungicide-sensitive”. (TIFF) [file pone.0262521.s002.tiff]

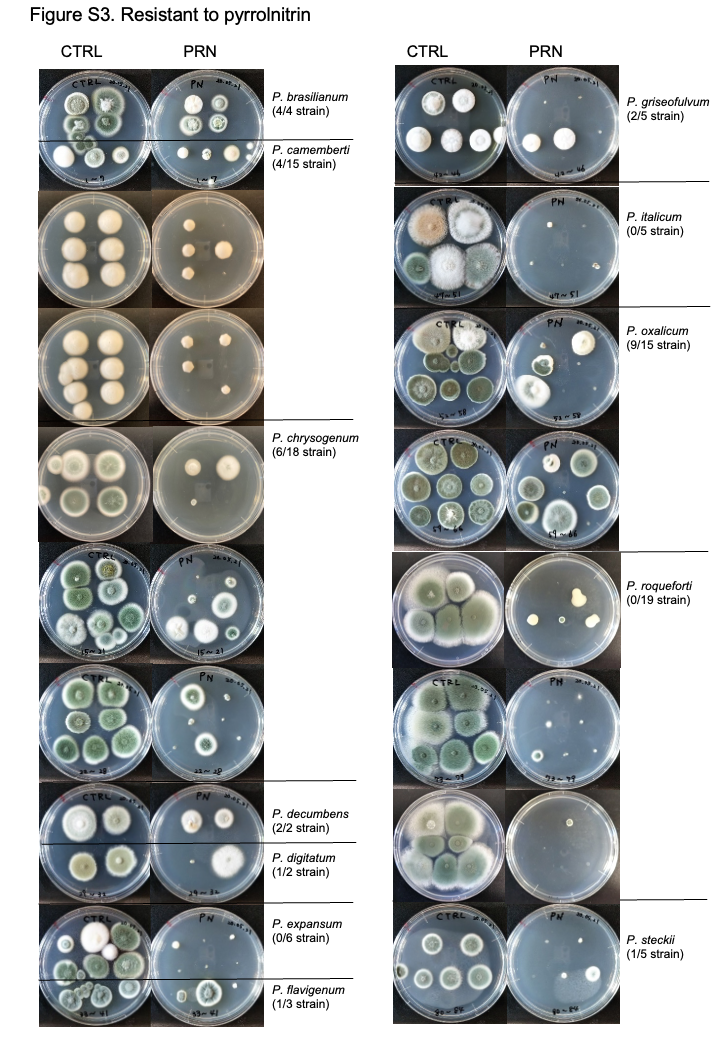

Supplement: S3 Fig — 99 Penicillium strains grown in PDA (left, CTRL) and PDA containing 0.05 μg/mL pyrrolnitrin (right, PRN). Colony growth rate in the presence of pyrrolnitrin ≥ 50%; “fungicide-resistant”, < 50%; “fungicide-sensitive”. (TIFF) [file pone.0262521.s003.tiff]
