# Supplementary material for: Wide distribution of resistance to the fungicides fludioxonil and iprodione in Penicillium species
Source: PLoS One. 2022 Jan 31;17(1):e0262521. doi: 10.1371/journal.pone.0262521 (PMC8803201; doi:10.1371/journal.pone.0262521)
Supplement: S1 Table — (DOCX) [file pone.0262521.s004.docx]

**S1 Table. PCR primers used in this study**

| **Name** | **Sequence** |
| --- | --- |
| Pchr_nikA_F | ATCCAAATCCATCCAAATTCCCGTCCCTTCTCACC |
| Pchr_ nikA _1 | ATGGCTACCGCGGACGAGACTCTCG |
| Pchr_ nikA _501 | GCAGGCCGAGGAGATAAGCAGCCAAAAG |
| Pchr_ nikA _1003 | GATCTTAGTCAGAAGATTGAGAGTCGGGCCCAAG |
| Pchr_ nikA _1501 | CTGACCACGCAGGTGCGTGAGATTGC |
| Pchr_ nikA _2001 | TGCTGGTACCTGGAAGGAAATCACCGAGGAC |
| Pchr_nikA_R2500 | CATCGGTGATTTCACCAAACGCGCGGAC |
| Pchr_ nikA _2501 | CTCTCAAGACATTGGCTGTCAAGGCCAATGAG |
| Pchr_ nikA _3001 | CGGGTGTTGTTCATCGACAAGGGTCAGAACGG |
| Pchr_ nikA _3501 | GGAAGCTGTCGAGGCTGTCAAGAAACGTC |
| Pchr_ nikA _R | CTTGTGCCGCGCTTGATATGCTCAGTAG |
| Poxa_nikA_F | TGGTTCCTTCTTTCGAATCGACCTGCTCCG |
| Poxa_nikA_1 | ATGGCTGGCGCGGACGAGACTCTC |
| Poxa_nikA_501 | AGATGAGACCGGCGCCACCCGTCTG |
| Poxa_nikA_1001 | ACATCATGGCCAAGAACCTCACGGATCAGGTG |
| Poxa_nikA_1501 | GCTACCGTCAATGACGTGGAAGGTACCTGGAAG |
| Poxa_nikA_2001 | ACTGAAAAAGGTCGCTCGCGATGTGGGAG |
| Poxa_nikA_R2500 | GATTCTCGGCCATGGTATTCACATCCTCGGTG |
| Poxa_nikA_2501 | CGAAGATTGAAGCCAACCGTATGGTCATTGAAAGC |
| Poxa_nikA_3001 | TGCGTGGTGAAGCTCGCTGATCAATCC |
| Poxa_nikA_3501 | CAACCAGCAGCTAGCGGTCAAGATACTCC |
| Poxa_nikA_R | CCACGTGTCGTACCAGAAAAGCCATAGAGGTC |
